# Sensory-Induced Human LTP-Like Synaptic Plasticity – Using Visual Evoked Potentials to Explore the Relation Between LTP-Like Synaptic Plasticity and Visual Perceptual Learning

**DOI:** 10.3389/fnhum.2021.684573

**Published:** 2021-06-25

**Authors:** Lilly Lengali, Johannes Hippe, Christoffer Hatlestad-Hall, Trine Waage Rygvold, Markus Handal Sneve, Stein Andersson

**Affiliations:** ^1^Department of Psychology, University of Oslo, Oslo, Norway; ^2^Department of Neurology, Oslo University Hospital, Oslo, Norway

**Keywords:** LTP, visual perceptual learning, visual evoked potentials, stimulus-selective response modulation, synaptic plasticity

## Abstract

**Objective:**

Stimulus-selective response modulation (SRM) of sensory evoked potentials represents a well-established non-invasive index of long-term potentiation-like (LTP-like) synaptic plasticity in the human sensory cortices. Although our understanding of the mechanisms underlying stimulus-SRM has increased over the past two decades, it remains unclear how this form of LTP-like synaptic plasticity is related to other basic learning mechanisms, such as perceptual learning. The aim of the current study was twofold; firstly, we aimed to corroborate former stimulus-SRM studies, demonstrating modulation of visual evoked potential (VEP) components following high-frequency visual stimulation. Secondly, we aimed to investigate the association between the magnitudes of LTP-like plasticity and visual perceptual learning (VPL).

**Methods:**

42 healthy adults participated in the study. EEG data was recorded during a standard high-frequency stimulus-SRM paradigm. Amplitude values were measured from the peaks of visual components C1, P1, and N1. Embedded in the same experimental session, the VPL task required the participants to discriminate between a masked checkerboard pattern and a visual “noise” stimulus before, during and after the stimulus-SRM probes.

**Results:**

We demonstrated significant amplitude modulations of VEPs components C1 and N1 from baseline to both post-stimulation probes. In the VPL task, we observed a significant change in the average threshold levels from the first to the second round. No significant association between the magnitudes of LTP-like plasticity and performance on the VPL task was evident.

**Conclusion:**

To the extent of our knowledge, this study is the first to examine the relationship between the visual stimulus-RM phenomenon and VPL in humans. In accordance with previous studies, we demonstrated robust amplitude modulations of the C1 and N1 components of the VEP waveform. However, we did not observe any significant correlations between modulation magnitude of VEP components and VPL task performance, suggesting that these phenomena rely on separate learning mechanisms implemented by different neural mechanisms.

## Introduction

Long-term potentiation (LTP) is suggested as one of the basic neurophysiological mechanisms for learning and memory and can be defined as the strengthening of synapses through repeated stimulation (Bliss and Lømo, 1973). In invasive animal models the LTP phenomena is thoroughly investigated in hippocampal slices (Cooke and Bliss, 2006), but also in other areas of the rodent brain, including the primary visual cortex (Heynen and Bear, 2001). Due to methodological limitations, the transition to human models explaining LTP have proven difficult. However, initiated by Teyler et al. (2005), non-invasive EEG-derived methods have been developed to study LTP-like phenomena in humans, targeting changes in sensory evoked potential amplitudes following high-frequency sensory stimulation (e.g., Teyler et al., 2005; Spriggs et al., 2017; Rygvold et al., 2020) or prolonged sensory stimulation (e.g., Normann et al., 2007; Valstad et al., 2020). The experimental procedure involving the modulation of sensory evoked potentials which is regarded as a valid assay of LTP-like plasticity phenomena, is commonly referred to as the stimulus-selective response modulation (SRM) paradigm (Rygvold et al., 2020).

The majority of research using the experimental SRM procedure has focused on the modulation of visual evoked potentials (VEPs). Although studies have targeted various components of the VEP waveform, amplitude alterations of the C1, P1, and N1/N1b are the most consistently reported findings. The SRM effect is suggested to reflect LTP-like plasticity, as the modulation effect evident in SRM paradigms shares several features with LTP in animal models, including input-specificity, longevity, and potentially also cooperativity (Cooke and Bear, 2014). Importantly, animal studies have reported that pharmacological blocking of *N*-Methyl-D-aspartic acid (NMDA) and α-amino-3-hydroxy-5-methyl-4-isoxazolepropionic acid (AMPA) receptor function inhibits the SRM effect (Cooke and Bear, 2014), suggesting that SRM-induced plasticity modulations of neural compounds in the visual sensory pathway also involves interactions between LTP processes and other plasticity mechanisms. In humans, differences in SRM magnitude are evident between age groups, drawing parallels to the age-related decline in LTP observed in animal studies (Spriggs et al., 2017; Abuleil et al., 2019). However, recently, it has been proposed that age-dependency is limited to a subset of the VEP components (Valstad et al., 2020). This latter study also reported significant modulation of several VEP components after prolonged visual stimulation and showed that the retention patterns of the N1b and N1 closely resemble LTP-like mechanisms.

Although our understanding of the SRM mechanisms has increased over the past two decades, it remains unclear how this form of LTP-like synaptic plasticity is related to other basic learning mechanisms, such as perceptual learning. Visual perceptual learning (VPL) refers to the improvement in visual perceptive ability as the result of learning (Bruns and Watanabe, 2019), putatively reflecting neural plasticity primarily in the visual system, but also association cortices and networks (Dosher and Lu, 2017). Importantly, LTP is suggested as the neural substrate of VPL, as several similarities between LTP and VPL are evident (Aberg and Herzorg, 2012). Consequently, animal research on SRM shed light on the similarities between changes in the sensory evoked potentials following HFS and perceptual learning (Frenkel et al., 2006; Cooke and Bear, 2014). Despite evidence for an association between LTP and perceptual learning in rodents (Sale et al., 2011) and the observed similarities between LTP mechanisms and perceptual learning, few studies have investigated this relationship in humans. In one study, Bao et al. (2010) observed an association between VPL and C1 modulation. Interestingly, the C1 peak manifests rapidly after stimulus onset and originates from cortical areas involved in early visual processing, suggesting a connection between perceptual learning and plasticity in the primary visual areas. Furthermore, studies suggest that visual high-frequency stimulation (HFS), similar to how it is implemented in the SRM experimental paradigm, leads to improvement of performance on visual tasks (Beste et al., 2011; Marzoll et al., 2018).

In the present study, we aimed to investigate the relationship between LTP*-*like plasticity indexed by amplitude changes in VEPs following HFS in the SRM framework, and performance on a VPL task. First, we expected to corroborate previous SRM studies, demonstrating significant modulation of VEP components following HFS. Second, we expected to observe significant associations between the magnitude of VEP component amplitude modulations and VPL task performance. Such associations might contribute to a broader understanding of how perceptual experience-dependent changes are related to neuroplastic processes. Moreover, the present study may contribute to an increased understanding of LTP-like plasticity and its importance in understanding human memory and learning.

## Materials and Methods

### Participants

Forty-two healthy volunteers (22 females, mean age 23.6 ± 4.08 years) participated after providing informed written consent. All participants were screened by personal interview, and participants reporting drug abuse during the past 6 months, history of neurological or psychiatric illness, or use of psycho-active medications were excluded. All participants reported normal or corrected-to-normal vision. Two subjects were excluded from the VEP and VPL analyses due to poor EEG data quality and invalid VPL task execution, respectively. This resulted in a VEP sample of 40 (21 females, mean age 23.7 ± 4.1), a VPL sample of 40 (21 females, mean age 23.6 ± 4.1), and a combined sample to investigate possible associations between the two, of 38 (20 females, mean age 23.7 ± 4.1 years). The project was approved by an internal ethical committee at the Department of Psychology, University of Oslo, Norway.

### Stimulus-Selective Response Modulation Paradigm

**FIGURE 1:**
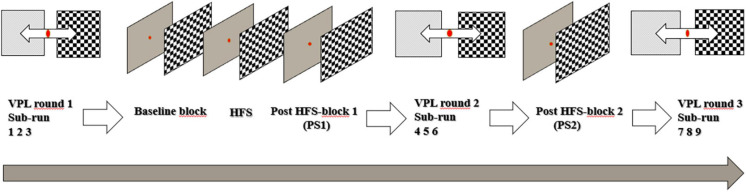
Timeline of the experimental setup.

As displayed in Figure 1, the stimulus-SRM VEP paradigm consisted of a baseline block, a HFS block, and two post-HFS blocks (PS1 and PS2). The visual stimulus was a reversing checkerboard pattern, displayed with maximum contrast. Each “check” of the stimulus covered 1 visual degree (spatial frequency = 1 cycle/degree). Both the baseline and the post-HFS blocks featured two sets of 40 stimulus reversals, separated by a brief interval of gray screen. The PS1 block followed directly after the HFS, whilst PS2 followed the second round of the VPL task (see Figure 1). Each stimulus reversal was presented with a stimulus onset asynchrony (SOA) randomly selected in the range 500–1,500 ms (mean reversal rate: 1 Hz). The participants were instructed to fixate on a red dot centered on the screen, and to ensure task compliance, the participants were asked to press a button whenever the dot changed color from red to green. Five such response cues were pseudo-randomly interspersed with the trials in each of the baseline and post-HFS blocks. These target stimuli were not included in the averaged VEP peak measurements. All stimulus blocks were separated by 30 s of gray screen. For the 120 s HFS block, the stimulus reversal frequency was locked to ∼8.55 Hz (customized to fit the monitor refresh frequency at 60 Hz), which corresponds to a SOA of ∼0.143 s. During the experiment, participants were seated with a chinrest, placed 57 cm away from 24″ LCD screen (BenQ, model ID: XL2420-B). The SRM paradigm was programmed and presented using Psychtoolbox-3 (Kleiner et al., 2007) in MATLAB (version 2015a; MathWorks, Natick, MA, United States).

### EEG Acquisition and Preprocessing

EEG signals were recorded using a 64-channel BioSemi ActiveTwo System (BioSemi, B.V., Amsterdam) with Ag-AgCl electrodes (sample rate: 1,024 Hz). Positioning of scalp electrodes was done in accordance with the extended 10–20 system (Oostenveld and Praamstra, 2001), with the addition of four electrodes externally around the eyes (LO1, LO2, IO2, and SO2). Preprocessing of EEG data was conducted using functions from EEGLAB (Delorme and Makeig, 2004; Delorme et al., 2012) on MATLAB (version 2017b). The raw EEG signals were re-referenced to the average of all signals, leaving out low quality signals. Then, the signals were filtered, using a band-pass with a lower limit of 1 Hz and an upper limit of 40 Hz (EEGLAB default settings). By evaluating the correlation between each signal and its spherically interpolated reconstruction, noisy signals were identified, and signals with a correlation below 0.75 were rejected. To identify and remove ocular artifacts, the signals were decomposed with the Second Order Blind Identification (SOBI; Belouchrani et al., 1993) algorithm, and categorized with ICLabel (Pion-Tonachini et al., 2019). Then, the signals were partitioned into 500 ms epochs (100 ms pre-stimulus to 400 ms post-stimulus). Epochs containing absolute amplitude values above 50 μV or relative abnormal amplitude patterns (EEGLAB function *pop_ jointprob*) in either O1, Oz, O2, or POz were rejected. Finally, VEP quality was evaluated by inspection of single epoch- and average epoch plots for the average of the signals from O1, Oz, and O2.

### Visual Evoked Potential Components Peak Measurement

Visual evoked potential components C1, P1, and N1 were defined as the first negative peak, first positive peak, and second negative peak, respectively. Amplitude values for each component was measured after a baseline correction of the VEP waveform (100 ms pre-stimulus to stimulus onset).

### Visual Perceptual Learning

The VPL task required participants to discriminate between a masked checkerboard pattern and a visual “noise” stimulus. The centers of the two equally sized stimuli (5 visual degrees, spatial frequency = 1 cycle/degree) were displayed horizontally aligned 10 visual degrees to either side of the screen’s vertical midline. The task employed an adaptive staircase routine (adopted from Psychtoolbox Quest), where the degree of task difficulty was calibrated according to performance on the five initial trials in each sub-run separately. The participants were instructed to fixate on a red dot in the center of the screen. The checkerboard pattern with graded masking, generated by combining white noise and checkerboard stimuli, and the visual “white noise” stimulus were shown simultaneously (randomly left/right) at each side of the fixation point. Manual response was given by pressing the right or left keyboard arrow key to indicate the side of the noise-embedded checkerboard stimulus presentation. The amount of checkerboard signal added to the noise varied in a participant-response dependent fashion and was controlled by the Quest staircase routine. The aim of the staircase routine was to find the proportion of signal (relative to noise) that produced a correct detection 75% of the time. The first five trials of a run were displayed with fixed proportion of signal [0.5 0.2 0.1 0.05 0.025], to ensure participant compliance. The VPL stimuli were presented with a SOA of 3,500 ms (mean presentation rate: ∼0.29 Hz). Each round of the VPL task was made up by 120 visual discrimination tasks, equally partitioned into three sub-runs consisting of 40 trials. In total, participants completed three rounds of the task, with a total of nine sub-runs. The three rounds were implemented before the VEP baseline block, between the VEP post-stimulation blocks 1 and 2, and after the VEP post-stimulation block 2 (Figure 1).

The average threshold level for each VPL round was based on the threshold levels of the three separate sub-runs, which together constituted one round of VPL. The average threshold level was defined as the participant’s average ability to separate a noise-embedded checkerboard pattern from pure noise with a 75% chance of success.

### Statistical Analyses

To investigate modulation of the VEP components of interest (C1, P1, and N1), change in amplitude levels (μV) from baseline block to PS1 and PS2 were analyzed using repeated measures ANOVAs. *Post hoc* pairwise comparisons, using paired samples *t*-tests, were done between baseline and PS1, baseline and PS2, and PS1 and PS2. Learning effects of the VPL paradigm was examined by comparing the average threshold levels between all rounds of the VPL task using pairwise comparisons (paired samples *t*-tests). Relationships between modulation of VEP components and VPL task performance were analyzed using Pearson linear correlations. In the exploratory effort of delineating possible associations between the SRM effect and VPL task performance, the linear correlation coefficient was computed between amplitude changes from baseline to PS1 for VEP components C1 and N1 and the difference in average threshold levels between round 2 and 1, and the best sub-run threshold level of the second round, in the VPL paradigm.

For the repeated measures ANOVA, the effect size estimate *partial eta squared* ($\eta_{\text{p}}^{2}$) is reported. $\eta_{\text{p}}^{2}$ is defined as the proportion of variance that can be explained by the difference between measurements at baseline and post-stimulation level (Lakens, 2013). For the pairwise comparisons, effect sizes are reported as Cohens *d*. In the following, *d* is calculated in accordance with Dunlap et al. (1996). In the following *p-*values will be reported uncorrected. Additionally, *t*-test *p*-values that survive correction for multiple comparisons using the Bonferroni method will be specified. Uncorrected *p*-values are presented in tables. All statistical analyses were conducted using IBM SPSS for Windows (version 26.0, IBM, Armont, NY, United States).

## Results

### Modulation of VEP-Components

**FIGURE 2:**
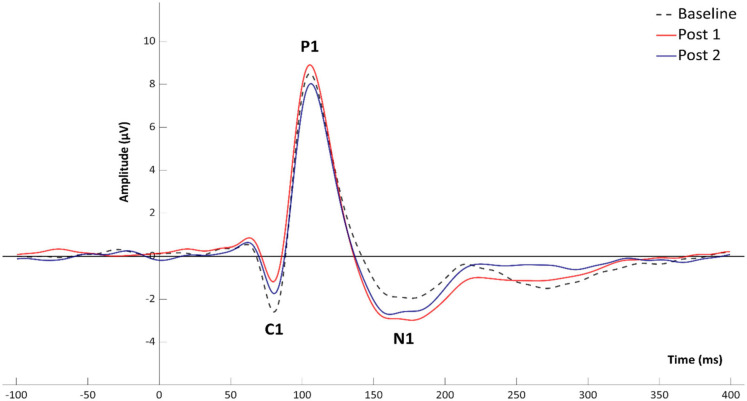
Mean amplitudes for VEP components C1, P1, and N1 at baseline and following HFS.

**FIGURE 3:**
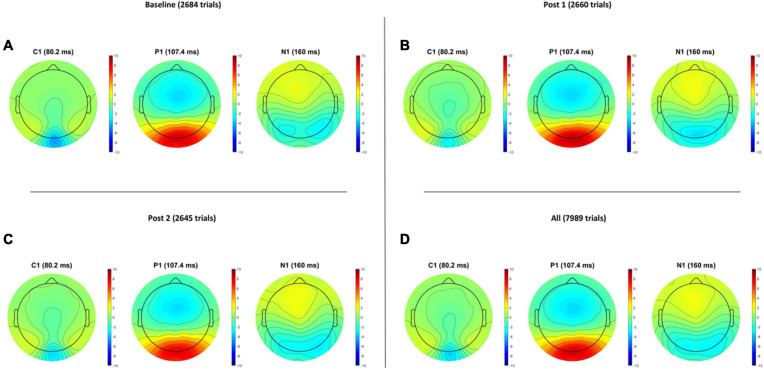
Visual evoked potential amplitude topographies for Baseline **(A)**, post HFS block 1 **(B)**, post HFS block 2 **(C)**, and all post HFS blocks **(D)**.

Grand average VEP waveforms are shown in Figure 2 with amplitude topgraphies visualized in Figure 3. As illustrated in Table 1, analyses of VEP modulation (repeated measures ANOVA) indicated a significant main effect of block in both components C1 [*F*(2.78) = 26.181, *p* < 0.001, $\eta_{\text{p}}^{2}$ = 0.40] and N1 [*F*(2.78) = 8.358, *p* = 0.001, $\eta_{\text{p}}^{2}$ = 0.17]. For P1, a trend-significant effect of block was observed [*F*(2.78) = 3.021, *p* = 0.054, $\eta_{\text{p}}^{2}$ = 0.07]. *Post hoc* pairwise comparisons showed significant modulation effects from baseline to PS1 for C1 [*t*(39) = −6.192, *p* < 0.001, *d* = −0.382] and N1 [*t*(39) = 3.986, *p* < 0.001, *d* = 0.258]. Furthermore, significant change was found from baseline to PS2 for both C1 [*t*(39) = −5.198, *p* < 0.001, *d* = −0.277] and N1 [*t*(39) = 2.306, *p* = 0.026, *d* = 0.169]. The P1 amplitude changes from baseline to PS1 [*t*(39) = −0.990, *p* = 0.328, *d* = −0.079] and from baseline to PS2 [*t*(39) = 1.330, *p* = 0.191, *d* = −0.107] were non-significant. The amplitude changes in C1 from baseline to PS1 and PS2, and in N1 from baseline to PS1, survive Bonferroni correction [*p* < 0.0125]; however, the change in N1 amplitude from baseline to PS2 does not.

**TABLE 1:** Mean (SD) amplitudes for visual evoked potential (VEP) components C1, P1, and N1 at baseline and following high frequency stimulation (HFS).

VEP component (*n* = 40)	Amplitude (mV) mean (SD)
**C1**	
Baseline	−3.239 (3.73)
Post HFS1	−1.836 (3.47)
Post HFS2	−2.207 (3.67)
**P1**	
Baseline	9.223 (3.73)
Post HFS1	9.533 (4.02)
Post HFS2	8.823 (3.45)
**N1**	
Baseline	−3.536 (3.26)
Post HFS1	−4.375 (3.21)
Post HFS2	−4.075 (3–06)

### Visual Perceptual Learning: Change in Average Threshold Levels

**FIGURE 4:**
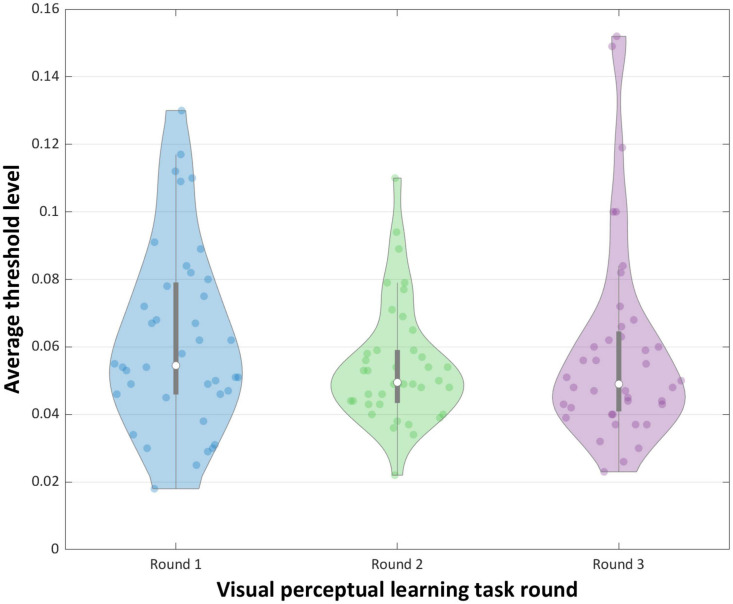
Mean performance (threshold levels) for the VPL learning task.

As illustrated in Figure 4 and analyzed in Table 2, repeated measures ANOVA showed a non-significant main effect of round [*F*(2.78) = 2.552, *p* = 0.084, $\eta_{\text{p}}^{2}$ = 0.06]. Further analyzes using *post hoc* pairwise comparisons indicated significant difference in average threshold levels between rounds 1 and 2 [*t*(39) = 2.354, *p* = 0.024, *d* = 0.315], but no significant difference between rounds 1 and 3, and between rounds 2 and 3. However, the observed significant difference between the average threshold level in rounds 1 and 2 did not survive a conservative Bonferroni correction [*p* > 0.016].

**TABLE 2:** Mean (SD) performance (threshold level) for the visual perceptual learning task (VPL).

Visual perceptual learning (*n* = 40)	Threshold level mean (SD)	Mean treshhold level range
Round 1 (sub-run 1–3)	0.062 (0.03)	0.122
Round 2 (sub-run 4–6)	0.054 (0.02)	0.088
Round 3 (sub-run 7–9)	0.058 (0.03)	0.128

### Correlations Between VEP-Modulation and Visual Perceptual Learning

Contrary to the theory-driven hypothesis, no significant correlations were observed between the VPL task performance and the modulation magnitude of VEP components.

## Discussion

The aims of the present study were to corroborate previous studies of VEP modulation within the SRM framework, and to investigate if these LTP-like plasticity processes can predict performance on a VPL task. First, we replicated previous studies of VEP modulation, demonstrating significant amplitude changes in VEP components C1 and N1 from baseline to both post-stimulation probes. The P1 amplitude, however, did not show significant increase following HFS Second, in the VPL task, we observed a significant reduction in the average threshold levels from the first to the second round. Finally, in contrast to our hypothesis, no significant association between LTP-like plasticity and VPL task performance were evident.

### Corroboration of the SRM Paradigm

In the present study, we demonstrated modulation of VEP components C1 and N1 following HFS. These findings add to previous studies demonstrating that high-frequency (Teyler et al., 2005; Spriggs et al., 2017; Rygvold et al., 2020) and prolonged (Normann et al., 2007; Valstad et al., 2020) sensory stimulation modulate several VEP component amplitudes. This modulation effect has been repeatedly demonstrated for components C1, P1, N1b, and N1, of which modulation of the N1/N1b complex is the most consistent finding (Teyler et al., 2005; Normann et al., 2007; Elvsåshagen et al., 2012; Spriggs et al., 2017; Zak et al., 2018; Valstad et al., 2020).

However, in contrast to some previous results, as well as the current hypothesis, we failed to replicate a corresponding modulation of the P1 peak amplitude. In a similar vein, Abuleil et al. (2019) found depotentiation in younger participants, whereas Valstad et al. (2020) report an association between P1 modulation and increasing age. The current sample was young (mean age 23.7), possibly explaining the absence of a P1 amplitude modulation. However, this effect of age has not been reported in other studies (e.g., Teyler et al., 2005; Normann et al., 2007; Elvsåshagen et al., 2012), suggesting that the SRM effect is not necessarily a phenomenon that can be generalized across individuals (Abuleil et al., 2019). This notion is further supported by the observation of sex differences in amplitude modulation magnitude (Valstad et al., 2020), a finding which may be related to systematic sex differences in the shape of cortical convolutions in the brain (Luders et al., 2004). Importantly, anatomical differences can potentially affect ERP amplitude measures (Valstad et al., 2020), making generalization challenging.

Furthermore, generalizing the SRM effect across stimulus parameters present challenges. This was demonstrated by Abuleil et al. (2019), who used a slightly different stimulation protocol than previous studies of SRM. In contrast to other SRM studies, these authors used central field stimulation and onset-offset presentation of stimuli. With the inclusion of VPL discrimination tasks this study also presents stimuli somewhat different than previous SRM research. Therefore it is possible that the present finding of a non-modulated P1 amplitude from baseline to the post-stimulation blocks may be accounted for by the addition of the VPL discrimination tasks. Conversely, these findings might reflect the possibility that the SRM effect itself is a heterogenous phenomenon. This notion may be further supported by studies employing functional magnetic resonance imaging (fMRI). One such study found increased blood-oxygen-level dependent (BOLD) response in visual brain areas after sensory stimulation (Clapp et al., 2005), whereas another reported a reduction of BOLD response on the group level (Lahr et al., 2014). Upon investigation of each subject individually, the latter study also found that differential increases or reductions of the BOLD signals were evident after sensory stimulation.

Finally, the current modest sample size might play a role in the lack of P1 amplitude modulation, as the effect sizes in SRM studies are generally low. To further our understanding of the SRM effect, we encourage future studies to investigate the effect of various individual factors on sensory induced VEP modulation.

### Associations Between the SRM Effect and Learning: VEP-Modulation and VPL

Interestingly, in the VPL task, we observed an improvement in the ability to visually discriminate between the target and the noise stimulus after a brief period of training combined with HFS. Although no significant main effect of round was evident, we observed a significant difference in average threshold levels between the first and second rounds of the VPL tasks. Thus, our results support previous findings indicating that brief high-frequency visual stimulation might play a role in the enhancement of visuo-perceptual ability (Beste et al., 2011; Pegado et al., 2016; Marzoll et al., 2018). One interpretation of these findings is that the VPL paradigm has resulted in learning, considering the observed significant difference in average threshold levels between the first and second round. Nevertheless, exactly how HFS affects learning and how persistent the effect is, remains uncertain, and available research is limited. The present results indicate no significant change in performance from the second to the third round. Similar to Pegado et al. (2016) this study indicates a brief improvement of performance from pre- to post-stimulation. However, this contrasts the more persistent learning effects seen in other studies (Beste et al., 2011; Marzoll et al., 2018). The lack of significant change in performance from the second to the third round has no unambiguous explanation; however, visual fatigue (Pegado et al., 2016) or perceptual deterioration (”overtraining”; Ashley and Pearson, 2012) are candidate mechanisms. Similar phenomena might be present in our study and could thus explain the observed lack of significant change in VPL task performance. Furthermore, considering the observed difference in range between the VPL blocks (see Table 2), an alternative explanation for the lack of significant change in task performance might be a decline in attention. However, the level of attention was not controlled for in our study, leaving this issue to be addressed in future studies.

In contrast to our hypothesis, we did not observe correlations between changes in C1 nor N1, and measures of VPL task performance. These results diverge from previous findings in both humans (Bao et al., 2010) and animal models (Sale et al., 2011), and also partly contrast previous findings of associations between enhancement of VPL and HFS, in where the latter is similarly employed as in studies targeting LTP-like plasticity (Beste et al., 2011; Marzoll et al., 2018).

The LTP-like plasticity captured by the SRM phenomenon is linked to visual areas in the brain, both in humans (Çavuş et al., 2012; Valstad et al., 2020) and rodents (Frenkel et al., 2006). Regarding VPL, it has been debated whether VPL is best explained by changes in the brain’s primary visual areas, or if the change is better understood as driven by neural plasticity later in the visual processing hierarchy. Watanabe and Sasaki (2015) argue that understanding VPL as either an alteration in early or in late visual areas, is an oversimplification. They suggest that VPL occurs based on changes across multiple areas related to the processing of visual information; a view termed the dual plasticity model. Others understand perceptual learning as even more complex, made possible by plasticity across numerous brain systems, making existing models unfit to capture its complexity (Maniglia and Seitz, 2018). Moreover, others highlight the possible contribution of neural networks involving cortical and subcortical structures outside the visual cortex in perceptual processes and perceptual learning (e.g., Deluca et al., 2014; Baumann et al., 2015; Yu et al., 2016). Summarized, one could understand plasticity as a complex phenomenon, where different capacities for plasticity varies between different areas and systems in the brain. Thus, identifying possible connections between SRM and performance-based VPL might be a complex endeavor. The lack of significant correlations between VPL and VEP in this study might reflect that the SRM phenomenon possibly indexes plasticity early in the visual processing hierarchy, whereas mastering the VPL tasks relies relatively more on selective attention and thus plasticity in brain areas of sensory integration. Conversely, there is also the possibility that the association between VPL and LTP-like plasticity occurs subcortically, hence explaining the absence of significant correlations in this study. Instead, the non-association between the two investigated paradigms observed in this study could instead have to do with methodological limitations, such as the size and homogeneity of the sample, rather than plasticity not being a global phenomenon.

Finally, there are profound differences between the invasive studies of LTP operating at single cell level and the non-invasive studies of the LTP-like SRM effect dependent on distributed neural activity (Sanders et al., 2018). The studies demonstrating how individual variation seemingly affects the SRM effect (Abuleil et al., 2019; Valstad et al., 2020) indicate that the experimental SRM paradigm employed here, differ from invasive studies of LTP in terms of the accuracy in which plasticity is measured. Differences in modulation response patterns of the VEP components suggest that separate mechanisms of plasticity could be involved (Valstad et al., 2020). Further development and use of non-invasive methods are warranted to increase our understanding of the link between invasive LTP and non-invasive LTP-like phenomena.

### Limitations

There are a several potential limitations related to the employed paradigms and procedures. First, in the VPL paradigm, the degree of task difficulty is calibrated according to performance on the five initial trials in each sub-run separately. Consequently, errors performed during the first five trials affect the final difficulty range of the remaining 35 trials in each sub-run. It is unclear whether the inclusion of more trials before the difficulty calibration would provide more robust performance measures. Second, this study used average change in threshold level as an indication of VPL. The average threshold level is a numerical expression of the participant’s average ability to distinguish between the stimuli and masking noise with a 75% chance of success. Currently, there is no consensus whether average threshold levels constitute a valid measure of VPL, and more research is encouraged. Third, the absence of longevity of the VEP potentiation in our findings may be associated with the incorporation of VPL stimuli between the two post-tetanus SRM measurements. Clapp et al. (2012) found that a sequence of stimuli presented at 1 Hz could depotentiate an already potentiated response. However, in the present study, the VPL SOA was 3,500 ms (∼0.29 Hz), suggesting that depotentiation of the VEP is an unlikely explanation. Still, the lack of control regarding the possible effect of VPL stimuli on VEP potentiation should be considered a limitation. Fourth, it is argued that a main characteristic of VPL is input specificity, making the passage of established learning across tasks limited or non-viable (Fiorentini and Berardi, 1980; Fahle, 2005; Clapp et al., 2012). Considering the principle of input specificity, the presentation of VEP and VPL stimuli to different visual fields in this study could lead to activation of contrasting neuronal assemblies, and thus explain the lack of observed significant associations. Some evidence (e.g., Watanabe and Sasaki, 2015) support such an understanding. However, there is currently no consensus for an explanatory model for the neural mechanisms behind VPL (Gold and Watanabe, 2010). Importantly, recording EEG during the VPL task might contribute to the particular question of stimulus specificity, and is warranted in future studies. Finally, another possible shortcoming is the modest total of 120 VPL trials in each round. A design with a greater number of trials included in each sub-run could possibly have increased measurement precision, and led to a more valid learning effect measure. However, the trade-off between measurement precision and fatigue must be considered. Also, a larger sample with increased heterogeneity with regard to age, could possibly have improved the validity of the learning effect measure, and thereby be more suited for generalization.

## Conclusion

This study is the first to examine the relationship between LTP-like synaptic plasticity indexed by stimulus-SRM of VEPs and VPL in humans. In accordance with previous studies, we demonstrated robust amplitude modulations of the C1 and N1 components of the VEP waveform. However, contrary to hypotheses, we did not observe any significant correlations between the SRM measures and the VPL task performance, suggesting that these phenomena might possibly rely on separate learning mechanisms implemented by different neural mechanisms.

## Data Availability Statement

The raw data supporting the conclusions of this article will be made available by the authors, without undue reservation.

## Ethics Statement

The studies involving human participants were reviewed and approved by Internal Ethics Committee at Department of Psychology, University of Oslo, etikk@psykologi.uio.no. The patients/participants provided their written informed consent to participate in this study.

## Author Contributions

LL, JH, TR, MS, CH-H, and SA contributed to the design and development of the study protocol. LL and JH have been responsible for participant recruitment and data acquisition. LL, JH, CH-H, and SA contributed to the data processing and statistical analyses. All authors contributed to the final manuscript, including final approval of the submitted version and have agreed to be accountable for all aspects of the work.

## Conflict of Interest

The authors declare that the research was conducted in the absence of any commercial or financial relationships that could be construed as a potential conflict of interest.

## References

[B1] Aberg K. C. Herzorg M. H. (2012). About similar characteristics of visual perceptual learning and LTP. *Vision Res.* 61 100–106. 10.1016/j.visres.2011.12.013 22289647

[B2] Abuleil D. Mcculloch D. L. Thompson B. (2019). Older adults exhibit greater visual cortex inhibition and reduced visual cortex plasticity compared to younger adults. *Front. Neurosci.* 13:607. 10.3389/fnins.2019.00607 31249506 PMC6582629

[B3] Ashley S. Pearson J. (2012). When more equals less: overtraining inhibits perceptual learning owing to lack of wakeful consolidation. *Proc. Biol. Sci.* 279 4143–4147. 10.1098/rspb.2012.1423 22896650 PMC3441080

[B4] Bao M. Yang L. Rios C. He B. Engel S. A. (2010). Perceptual learning increases the strength of the earliest signals in visual cortex. *J. Neurosci.* 30 15080–15084. 10.1523/JNEUROSCI.5703-09.2010 21068313 PMC3073503

[B5] Baumann O. Borra R. J. Bower J. M. Cullen K. E. Habas C. Ivry R. B. (2015). Consensus paper: the role of the cerebellum in perceptual processes. *Cerebellum* 14 197–220. 10.1007/s12311-014-0627-7 25479821 PMC4346664

[B6] Belouchrani A. Abed-Meraim K. Cardoso J.-F. (1993). “Second-order blind separation of temporally correlated sources,” in *Proceedings of the International Conference of Digital Signal Processing* (Paphos: Citeseer), 346–351.

[B7] Beste C. Wascher E. Güntürkün O. Dinse H. R. (2011). Improvement and impairment of visually guided behavior through LTP- and LTD-like exposure-based visual learning. *Curr. Biol.* 21 876–882. 10.1016/j.cub.2011.03.065 21549600

[B8] Bliss T. V. Lømo T. (1973). Long−lasting potentiation of synaptic transmission in the dentate area of the anaesthetized rabbit following stimulation of the perforant path. *J. Physiol.* 232 331–356. 4727084 10.1113/jphysiol.1973.sp010273 PMC1350458

[B9] Bruns P. Watanabe T. (2019). Perceptual learning of task-irrelevant features depends on the sensory context. *Sci. Rep.* 9:1666. 30733577 10.1038/s41598-019-38586-8 PMC6367344

[B10] Çavuş I. Reinhart R. M. G. Roach B. J. Gueorguieva R. Teyler T. J. Clapp W. C. (2012). Impaired visual cortical plasticity in schizophrenia. *Biol. Psychiatry* 71 512–520. 10.1016/j.biopsych.2012.01.013 22364738 PMC3292767

[B11] Clapp W. C. Hamm J. P. Kirk I. J. Teyler T. J. (2012). Translating long-term potentiation from animals to humans: a novel method for noninvasive assessment of cortical plasticity. *Biol. Psychiatry* 71 496–502. 10.1016/j.biopsych.2011.08.021 21974785 PMC3253317

[B12] Clapp W. C. Zaehle T. Lutz K. Marcar V. L. Kirk I. J. Hamm J. P. (2005). Effects of long-term potentiation in the human visual cortex: a functional magnetic resonance imaging study. *Neuroreport* 16 1977–1980. 10.1097/00001756-200512190-00001 16317337

[B13] Cooke S. F. Bear M. F. (2014). How the mechanisms of long-term synaptic potentiation and depression serve experience-dependent plasticity in primary visual cortex. *Philos. Trans. R. Soc. Lond. B Biol. Sci.* 369:20130284. 10.1098/rstb.2013.0284 24298166 PMC3843896

[B14] Cooke S. F. Bliss T. V. P. (2006). Plasticity in the human central nervous system. *Brain*, 129 1659–1673. 10.1093/brain/awl082 16672292

[B15] Delorme A. Makeig S. (2004). EEGLAB: an open source toolbox for analysis of single-trial EEG dynamics including independent component analysis. *J. Neurosci. Methods* 134 9–21. 10.1016/j.jneumeth.2003.10.009 15102499

[B16] Delorme A. Palmer J. Onton J. Oostenveld R. Makeig S. (2012). Independent EEG sources are dipolar. *PLoS One* 7:e30135. 10.1371/journal.pone.0030135 22355308 PMC3280242

[B17] Deluca C. Golzar A. Santandrea E. Gerfo E. L. Eštočinová J. Moretto G. (2014). The cerebellum and visual perceptual learning: evidence from a motion extrapolation task. *Cortex* 58 52–71. 10.1016/j.cortex.2014.04.017 24959702

[B18] Dosher B. Lu Z. L. (2017). Visual perceptual learning and models. *Annu. Rev. Vis. Sci.* 3 343–363. 28723311 10.1146/annurev-vision-102016-061249 PMC6691499

[B19] Dunlap W. P. Cortina J. M. Vaslow J. B. Burke M. J. (1996). Meta-analysis of experiments with matched groups or repeated measures designs. *Psychol. Methods* 1 170–177. 10.1037/1082-989X.1.2.170

[B20] Elvsåshagen T. Moberget T. Bøen E. Boye B. Englin N. O. A. Pedersen P. (2012). Evidence for impaired neocortical synaptic plasticity in bipolar II disorder. *Biol. Psychiatry* 71 68–74. 10.1016/j.biopsych.2011.09.026 22036034

[B21] Fahle M. (2005). Perceptual learning: specificity versus generalization. *Curr. Opin. Neurobiol.* 15 154–160. 15831396 10.1016/j.conb.2005.03.010

[B22] Fiorentini A. Berardi N. (1980). Perceptual learning specific for orientation and spatial frequency. *Nature* 287 43–44. 7412873 10.1038/287043a0

[B23] Frenkel M. Y. Sawtell N. B. Diogo A. C. M. Yoon B. Neve R. L. Bear M. F. (2006). Instructive effect of visual experience in mouse visual cortex. *Neuron* 51 339–349. 10.1016/j.neuron.2006.06.026 16880128

[B24] Gold J. I. Watanabe T. (2010). Perceptual learning. *Curr. Biol.* 20 46–48. 10.1016/j.cub.2009.10.006 20129034 PMC3821996

[B25] Heynen A. J. Bear M. F. (2001). Long-term potentiation of thalamocortical transmission in the adult visual cortex in vivo. *J. Neurosci.* 21 9801–9813. 11739588 10.1523/JNEUROSCI.21-24-09801.2001 PMC6763060

[B26] Kleiner M. Brainard D. Pelli D. Ingling A. Murray R. Broussard C. (2007). What’s new in psychtoolbox-3. *Perception* 36 1–16.

[B27] Lahr J. Peter J. Bach M. Mader I. Nissen C. Normann C. (2014). Heterogeneity of stimulus-specific response modification—an fMRI study on neuroplasticity. *Front. Hum. Neurosci.* 8:695. 10.3389/fnhum.2014.00695 25249962 PMC4157554

[B28] Lakens D. (2013). Calculating and reporting effect sizes to facilitate cumulative science: a paractical primer for t-tests and ANOVAs. *Front. Psychol*. 4:863. 24324449 10.3389/fpsyg.2013.00863 PMC3840331

[B29] Luders E. Narr K. Thompson P. Rex D. E. Jancke L. Steinmetz H. (2004). Gender differences in cortical complexity. *Nat. Neurosci.* 7 799–800. 10.1038/nn1277 15338563

[B30] Maniglia M. Seitz A. R. (2018). Towards a whole brain model of perceptual learning. *Curr. Opin. Behav. Sci.* 20 47–55. 10.1016/j.cobeha.2017.10.004 29457054 PMC5810967

[B31] Marzoll A. Saygi T. Dinse H. R. (2018). The effect of LTP- and LTD-like visual stimulation on modulation of human orientation discrimination. *Sci. Rep.* 8:16156. 10.1038/s41598-018-34276-z 30385849 PMC6212525

[B32] Normann C. Schmitz D. Fürmaier A. Döing C. Bach M. (2007). Long-term plasticity of visually evoked potentials in humans is altered in major depression. *Biol. Psychiatry* 62 373–380. 10.1016/j.biopsych.2006.10.006 17240361

[B33] Oostenveld R. Praamstra P. (2001). The five percent electrode system for high resolution EEG and ERP measurements. *Clin. Neurophysiol.* 112 713–719. 10.1016/S1388-2457(00)00527-7 11275545

[B34] Pegado F. Vankrunkelsven H. Steyaert J. Boets B. De Beeck H. O. (2016). Exploring the use of sensorial LTP/LTD-Like stimulation to modulate human performance for complex visual stimuli. *PLoS One* 11:e0158312. 10.1371/journal.pone.0158312 27341210 PMC4920386

[B35] Pion-Tonachini L. Kreutz-Delgado K. Makeig S. (2019). ICLabel: an automated electroencephalographic independent component classifier, dataset, and website. *Neuroimage* 198 181–197. 10.1016/j.neuroimage.2019.05.026 31103785 PMC6592775

[B36] Rygvold T. W. Hatlestad−Hall C. Elvsåshagen T. Moberget T. Andersson S. (2020). Do visual and auditory stimulus−specific response modulation reflect different mechanisms of neocortical plasticity? *Eur. J. Neurosci.* 53 1072–1085. 10.1111/ejn.14964 32897598

[B37] Sale A. De Pasquale R. Bonaccorsi J. Pietra G. Olivieri D. Berardi N. (2011). Visual perceptual learning induces long-term potentiation in the visual cortex. *Neuroscience* 172 219–225. 21056088 10.1016/j.neuroscience.2010.10.078

[B38] Sanders P. J. Thompson B. Corballis P. M. Maslin M. Searchfield G. D. (2018). A review of plasticity induced by auditory and visual tetanic stimulation in humans. *Eur. J. Neurosci*. 48 2084–2097. 10.1111/ejn.14080 30025183

[B39] Spriggs M. J. Cadwallader C. J. Hamm J. P. Tippett L. J. Kirk I. J. (2017). Age-related alterations in human neocortical plasticity. *Brain Res. Bull.* 130 53–59. 10.1016/j.brainresbull.2016.12.015 28043855

[B40] Teyler T. J. Hamm J. P. Clapp W. C. Johnson B. W. Corballis M. C. Kirk I. J. (2005). Long−term potentiation of human visual evoked responses. *Eur. J. Neurosci.* 21 2045–2050. 10.1111/j.1460-9568.2005.04007.x 15869500 PMC1226326

[B41] Valstad M. Moberget T. Roelfs D. Slapø N. B. Timpe C. M. F. Beck D. (2020). Experience-dependent modulation of the visual evoked potential: testing effect sizes, retention over time, and associations with age in 415 healthy individuals. *Neuroimage* 223:117302. 10.1016/j.neuroimage.2020.117302 32828930

[B42] Watanabe T. Sasaki Y. (2015). Perceptual learning: toward a comprehensive theory. *Annu. Rev. Psychol.* 66 197–221. 25251494 10.1146/annurev-psych-010814-015214 PMC4286445

[B43] Yu Q. Zhang P. Qiu J. Fang F. (2016). Perceptual learning of contrast detection in the human lateral geniculate nucleus. *Curr. Biol.* 26 3176–3182. 10.1016/j.cub.2016.09.034 27839973

[B44] Zak N. Moberget T. Bøen E. Boye B. Waage T. R. Dietrichs E. (2018). Longitudinal and cross-sectional investigations of long-term potentiation-like cortical plasticity in bipolar disorder type II and healthy individuals. *Transl. Psychiatry* 8:103. 10.1038/s41398-018-0151-5 29795193 PMC5966393

